# Fast splice site detection using information content and feature reduction

**DOI:** 10.1186/1471-2105-9-S12-S8

**Published:** 2008-12-12

**Authors:** AKMA Baten, SK Halgamuge, BCH Chang

**Affiliations:** 1 Biomechanical Engineering Research Group, Department of Mechanical Engineering, Melbourne School of Engineering, The University of Melbourne, Victoria 3010, Australia; 2 Institute of Plant and Microbial Biology, Academia Sinica, Taiwan

## Abstract

**Background:**

Accurate identification of splice sites in DNA sequences plays a key role in the prediction of gene structure in eukaryotes. Already many computational methods have been proposed for the detection of splice sites and some of them showed high prediction accuracy. However, most of these methods are limited in terms of their long computation time when applied to whole genome sequence data.

**Results:**

In this paper we propose a hybrid algorithm which combines several effective and informative input features with the state of the art support vector machine (SVM). To obtain the input features we employ information content method based on Shannon's information theory, Shapiro's score scheme, and Markovian probabilities. We also use a feature elimination scheme to reduce the less informative features from the input data.

**Conclusion:**

In this study we propose a new feature based splice site detection method that shows improved acceptor and donor splice site detection in DNA sequences when the performance is compared with various state of the art and well known methods.

## Background

Over the past decades, the scientific community has experienced a major growth in numbers of sequence data. With the emergence of novel and efficient sequencing technology, DNA sequencing is now much faster. Sequencing of several genomes including the human genome have been completed successfully. This massive amount of sequence data demands sophisticated tools for the analysis of data.

Identifying genes accurately is one of the most important and challenging tasks in bioinformatics and it requires the prediction of the complete gene structure. Identification of splice sites is the core component of eukaryotic gene finding algorithms. Their success depends on the precise identification of the exon-intron structure and the splice sites. Most of the eukaryotic protein coding genes are characterized by exons and introns. Exons are the protein coding portion of a gene and they are segmented with intervening sequences of introns. The border between an exon and an intron is known as the splice site. The splice site upstream of an intron is called the donor splice site (in the direction 5' to 3') and one that is downstream of an intron is the acceptor splice site (in the direction 3' to 5'). The consensus sequence refers to the nucleotides, which are conserved or most frequently observed in a particular position. The acceptor and donor splice sites with consensus AG (corresponding to the end of an intron) and GT (corresponding to the beginning of an intron) dinucleotides respectively are known as canonical splice sites. Approximately 99% of the splice sites are canonical [1]. As AG and GT represent possible acceptor and donor splice sites, every AG and GT in a DNA sequence is a candidate acceptor or donor splice site and they need to be classified as either a real (true) splice site or a pseudo (false) splice site.

Over the years many computational methods have been proposed for the identification of splice sites. Most of those methods are designed to identify the apparent consensus AG and GT in the splicing junction. These methods can be largely classified into probabilistic methods [2-8], neural network and support vector machine methods [9-19], and methods based on discriminant analysis [20,21]. Neural networks and support vector machines (SVM) learn the complex features of neighbourhoods surrounding the consensus AG/GT dinucleotides by a complex non-linear transformation. Probabilistic models estimate position specific probabilities of splice sites by computing likelihoods of candidate signal sequences. The discriminant analysis uses several statistical measures to evaluate the presence of specific nucleotides, recognizing the splice sites without explicitly determining the probability distributions [18].

In DNA sequences, true consensus AG/GT dinucleotides are outnumbered by many false AG/GTs. However, nucleotides surrounding true AG/GTs show a certain nucleotide dependency and sequential relationship compared to those surrounding false AG/GTs. There are several methods which are particularly designed to capture this relationship and to identify true splice sites among numerous false ones. Weight matrix methods (WMM) and methods based on Markov models are popular methods of this category. WMM was successfully adopted in methods like NetPlantGene [22] and NNSplice [10]. Salzberg *et al. *and Zhang *et al. *[2,6], used a linear first order Markov model (MM1) also known as the weight array method (WAM) and they have achieved a good splice site prediction accuracy. MM1 only utilizes first order sequential relationship. It is desirable to use a higher order Markov model to capture the higher order and extended sequential relationship. However, the computational complexity increases polynomialy with the increase of the order of the Markov model, and also higher order Markov models require a large number of training samples. The maximal dependence decomposition (MDD) algorithm was proposed by Burge *et al*. [23] to overcome these limitations. MDD is a decision tree process and models the dependency between adjacent nucleotides. To take the advantages of both MDD and Markov models, Pertea *et al. *[4] proposed the GeneSplicer method which combines MDD and second order Markov models (MM2). GeneSplicer showed an improved splice site detection performance. More recently, Rajapakse *et al. *[17] proposed a complex splice site detection method by combining mostly second order Markov models with backpropagation neural networks (BPNN). This method showed an improved performance over GeneSplicer, however, BPNN is already computationally expensive and this method requires a larger sequence window. In contrast, a machine learning technique such as SVM has the advantage of inferring an optimal classifier from the training data. SVM has been used to classify splice site data with limited success [9,12,14-16].

Most of the existing splice site detection methods focused on the improvement of classification performance. However other studies suggest that, considering the increasing growth of sequence data, the focus of new methods should be towards developing faster methods [24-27]. In our previous work we showed an improved splice site detection performance by using several preprocessing methods including WMM0/MM0, WMM1, MM1 with SVM [18]. However, the training time and the number of input features to SVM is a major concern. SVM performs better when it is trained with the most important and meaningful features. So, the reduction of less important features may improve both the classification performance and training time of SVM. In this paper, we propose a feature selection strategy which reduces the less important features from the input data. We also combine the well studied information content method based on Shannon's information theory [28-30] and the Shapiro's score method [31] to extract meaningful information from sequence that can potentially identify splice sites. Our method showed an improved splice site detection performance when compared to the existing methods in terms of classification accuracy and training time.

## Results

### Classification performance comparison

Our hybrid algorithm combines several effective and informative input features with the state of the art support vector machine (SVM). To obtain the input features we employ information content method based on Shannon's information theory, Shapiro's score scheme, and Markovian probabilities. We also use the F-score feature elimination scheme to reduce the less informative features from the input data. We use the publicly available NN269 [10] splice site dataset to evaluate the performance of our method. The MM1 parameters are calculated from the dataset and F-score method (refer to the method section) is applied to reduce the number of MM1 parameters, which is referred as Reduced MM1 SVM method. We also calculate the information content and Shapiro's score from the dataset and use the proposed the IC Shapiro SVM method, which is a linear combination of information content and Shapiro's score. We compare the performance of our methods with MM1 SVM method as proposed [18]. To evaluate the classification performance we use several performance evaluation methods such as the sensitivity, specificity, receiver operating characteristics curve (ROC), and the area under ROC (AUC) as described in the method section.

**Figure 1:**
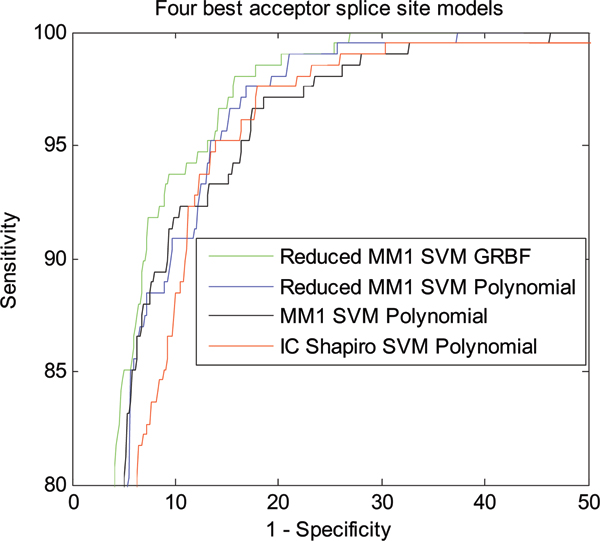
ROC curve showing the classification performance of different models for NN269 acceptor splice site data.

**Figure 2:**
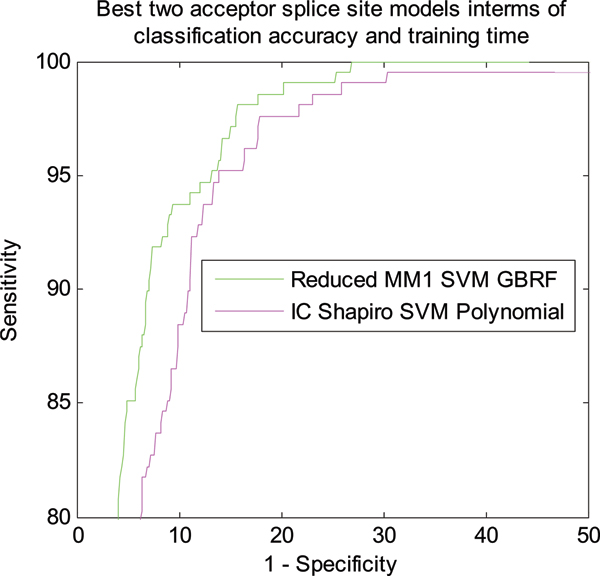
ROC curve showing the classification performance of best two models in terms of accuracy and training time for NN269 acceptor splice site data.

Figure 1 shows the classification performance of different models for NN269 acceptor splice site data. The performance of the proposed Reduced MM1 SVM and IC Shapiro SVM is compared with the original MM1 SVM model [18]. As shown in Figure 1, the Reduced MM1 SVM model with GRBF kernel produces the best classification performance for acceptor splice sites. Reduced MM1 SVM with polynomial kernel produces the second best performance. MM1 SVM with polynomial kernel method [18], produces the third best performance while the performance of IC Shapiro SVM with polynomial kernel is not as good as others. Even though, Reduced MM1 SVM with GRBF kernel shows the best classification performance, from the ROC curve we can see all the models perform very closely and hence, to get a better measure of the classification performance we calculated the AUC covered by each model from the ROC. Computational speed is another important issue for the algorithms applied in this problem. In this regard, we also calculate the training time required for each classification models. For all our simulations we used an Intel P4 3.2 GHz system with 1 GB RAM. Both the AUC and training time for each of the models are shown in Table 1. Figure 2 shows the best two models for acceptor splice site identification in terms of best accuracy (Reduced MM1 SVM with GRBF kernel) and best training time (IC Shapiro SVM with polynomial kernel).

**Table 1:** AUC and training time for different models for NN269 acceptor splice sites.

**Model**	**SVM kernel**	**AUC**	**Training time until convergence (hh.mm.ss)**
Reduced MM1 SVM (Best in terms of accuracy)	GRBF	**0.9741389**	**00.22.17**

Reduced MM1 SVM	Polynomial	0.9695822	00.10.48

MM1 SVM [18]	Polynomial	0.9674048	00.11.02

IC Shapiro SVM (Best In terms of Time)	Polynomial	**0.96287**	**00:01:18**

As shown in Table 1, Reduced MM1 SVM with GRBF kernel produces the best performance with an AUC area of 0.9741. Reduced MM1 SVM with polynomial kernel produces the second best performance with an AUC of 0.9695 while MM1 SVM with polynomial kernel [18] has an AUC of 0.9674. Though IC Shapiro SVM with polynomial kernel has an AUC of 0.9628, which is marginally worse than the best performing model, it produces the fastest training time. Table 2 shows the improvement of performance in terms of AUC and training time as compared to MM1 SVM Polynomial [18].

**Table 2:** AUC and training time improvement for different models compared to MM1-SVM method for NN269 acceptor splice sites.

**Model**	**SVM kernel**	**AUC**	**Training time until convergence (mm.ss)**	**Performance Improvement**	**Time Improvement**
MM1 SVM [18]	Polynomial	0.9674	11.02	-	-

Reduced MM1 SVM (Best in terms of accuracy)	GRBF	0.9741	22.17	0.69%	-101.96%

Reduced MM1 SVM	Polynomial	0.9695	10.48	0.2171%	2.11%

IC Shapiro SVM (Best In terms of Time)	Polynomial	0.9628	01:18	-0.4755%	88.21%

As shown in Table 2, the best acceptor splice site detection performance is produced by Reduced MM1 SVM with GRBF kernel which is 0.69% superior then MM1 SVM with polynomial kernel. However, Reduced MM1 SVM GRBF requires much longer training time (more than 100%) than MM1 SVM Polynomial. Reduced MM1 SVM Polynomial improves the performance by 0.21% and it also 2.11% faster than MM1 SVM Polynomial. Finally, IC Shapiro SVM Polynomial is just 0.47% worse then MM1 SVM Polynomial, however, it shows a significant improvement in the training time and is 88.21% faster than MM1 SVM Polynomial.

**Figure 3:**
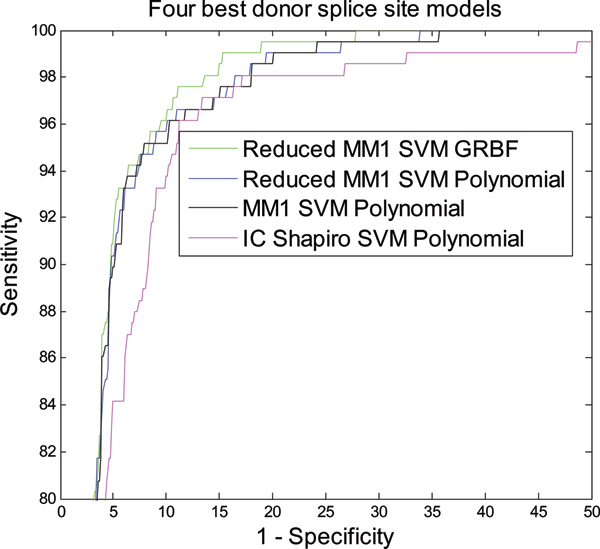
ROC curve showing the classification performance of different models for NN269 donor splice site data.

**Figure 4:**
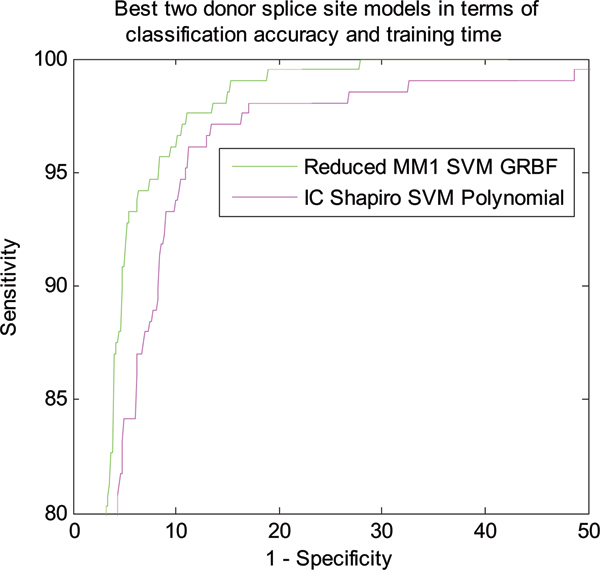
ROC curve showing the classification performance of best two models in terms of accuracy and training time for NN269 donor splice site data.

Figure 3 shows the classification performance of different models in terms of NN269 donor splice site dataset. The performance of all the models developed in this paper is compared with MM1 SVM Polynomial model [18]. As shown in Figure 3, the Reduced MM1 SVM model with GRBF kernel produces the best classification performance for donor splice sites. Reduced MM1 SVM with polynomial kernel produces the second best performance. Performance of all the models is very close except IC Shapiro SVM with polynomial kernel. The performance of the models shows the similar trend as that of acceptor splice site classification. We also calculate AUC and the training time required for each of the models which are shown in Table 3. Figure 4 shows the two best methods in terms of classification accuracy (Reduced MM1 SVM GRBF) and training time (IC Shapiro SVM Polynomial).

**Table 3:** AUC and training time for different models for NN269 donor splice sites.

**Model**	**SVM kernel**	**AUC**	**Training time until convergence (hh.mm.ss)**
Reduced MM1 SVM (Best in terms of accuracy)	GRBF	**0.9790232**	**00:20:04**

Reduced MM1 SVM	Polynomial	0.9764903	00:09:30

MM1 SVM	Polynomial	0.9761952	00:10:02

IC Shapiro SVM (Best In terms of Time)	Polynomial	**0.9665982**	**00:02:59**

As shown in Table 3, Reduced MM1 SVM with GRBF kernel is the best model for donor splice site classification with an AUC area of 0.9790. Reduced MM1 SVM Polynomial is marginally worse then Reduced MM1 SVM GRBF and produces the second best performance with an AUC of 0.9764 while MM1 SVM with polynomial kernel [18] has an AUC of 0.9761. Following the same trend, IC Shapiro SVM Polynomial also produces the fastest training time. Table 4 shows the improvement of performance in terms of AUC and training time as compared to MM1 SVM Polynomial [18].

**Table 4:** AUC and training time improvement for different models compared to MM1-SVM method for NN269 donor splice sites.

**Model**	**SVM kernel**	**AUC**	**Training time until convergence (mm.ss)**	**Performance Improvement**	**Time Improvement**
MM1 SVM	Polynomial	0.9761	10:02	-	-

Reduced MM1 SVM (Best in terms of accuracy)	GRBF	0.9790	20:04	0.297%	-100%

Reduced MM1 SVM	Polynomial	0.9764	09:30	0.0102%	5.31%

IC Shapiro SVM (Best In terms of Time)	Polynomial	0.9665	02:59	-0.9835%	70.26%

As shown in Table 4, Reduced MM1 SVM GRBF marginally produces the best acceptor splice site detection performance which is 0.29% superior then MM1 SVM with polynomial kernel. However, it requires much longer training time (more than 100%) than MM1 SVM Polynomial. Reduced MM1 SVM Polynomial performs almost equally as well as MM1 SVM Polynomial, though it is 5.31% faster. Finally, IC Shapiro SVM Polynomial is almost 1% worse then MM1 SVM Polynomial, however, it shows a significant improvement in the training time and is 70.26% faster than MM1 SVM Polynomial. All the parameters regarding the SVM implementations with GRBF and Polynomial kernels are provided in Additional file 1: Table S1.

## Discussions

One of the biological machineries involved in the splicing process is known as the Spliceosome, which binds in a splice site after determining the information available in that site. Information content and Shapiro's score are two well known methods to determine the information in splice sites [29,30]. Previously we have used other methods such as first order Markov model (MM1), first order weight matrix model (WMM1) and zero order Markov/Weight matrix model (MM0/WMM0) to capture such information [18]. However, our results in this paper show that information content and Shapiro's score are more capable of capturing more meaningful information than those methods we have used previously, which justifies the use of these methods to extract features. Our method based on information content and Shapiro's score is also proved to be much faster.

We also use the F-score feature ranking measure to select the most meaningful features and use it to eliminate less important features. We use the F-score measure to reduce MM1 parameters and when compared with MM1 SVM method [18], we find that Reduced MM1 SVM method based on reduced MM1 parameters performs better in terms of classification accuracy as shown in Figures 1, 2, 3, and 4. The performance of IC Shapiro SVM is marginally worse then the best performing methods. However, it shows much faster training time than others as IC Shapiro SVM uses much less number of features. This IC and Shapiro's score scheme and their integration as a set of features is an important step towards faster splice site identification and can be effectively used in the splice site detection for the whole genome where vast amount of sequences data is available. However, it is worthwhile to further investigate this method to improve its classification accuracy.

## Conclusion

Modern sequencing techniques can produce a massive amount of data in short time and the number of sequence data is almost exponentially increasing. Fast splice site detection is very useful when we consider the very large volumes of available data for the training and testing of a method. To cope with such a large volume of data we also need faster methods. The fast splice site detection method we propose in this paper can also be applied to identify other signals in the sequence such as promoters and translation initiation sites.

## Methods

### Proposed models

We propose several models for the identification of acceptor and donor splice sites. Corresponding to the two types of splice sites, the splice site classification problem is subdivided into two classification problems: acceptor splice site classification and donor splice site classification. Two separate models are constructed for the identification of acceptor splice sites and donor splice sites respectively.

All the proposed models consist of two phases. In phase one, sequence features are extracted, and in phase two, a support vector machine is trained with the selected features. Sequence features are extracted using first order Markov model (MM1), information content (IC), and Shapiro's score method (SS). The IC score and SS score for each splice site sequence are calculated and linearly combined together as one input to the SVM. The proposed models are listed in Table 5.

**Table 5:** Proposed models and their description.

**Model**	**Description**
Reduced MM1 SVM Polynomial	Only reduced MM1 parameters and SVM with polynomial kernel

Reduced MM1 SVM GRBF	Only reduced MM1 parameters and SVM with GRBF kernel

IC Shapiro SVM Polynomial	Information content, Shapiro's score and SVM with polynomial kernel

### Markov model

Markov model can be regarded as a finite state machine with Markov property. Let us consider a sequence of random variables *X*_1_, *X*_2_,..... *X*_*n *_which takes on values from a finite state space *A *= {*A*_1_, *A*_2_,... *A*_*n*_}. If the probability of transition from state *A*_*i *_at time *n *to state *A*_*j *_at time *n *+1 depends only on *A*_*i*_, and not any previous history of process, then the process is said to have the Markov property or to be a Markov model or Markov chain.

DNA sequences can be represented by a Markov chain where each nucleotide represents a state in the Markov chain and whose observed state variables are drawn from the alphabet Ω_*DNA *_= {*A*, *C*, *G*, *T*}. If we consider a sequence of length *l*: {*s*_1_, *s*_2_,...., *s*_*l*_}, where *s*_*i *_∈ {*A*, *C*, *G*, *T*}, ∀*i *∈ {1,...., *l*}, then the nucleotide *S*_*i *_is the outcome of the *i *th state variable of the Markov model, and state transition is only allowed from state *i *to its adjacent state *i *+1. Hence, the model consists of states ordered in a series. It evolves from state *s*_*i *_to *s*_*i*+1 _and emits symbols from the alphabet Ω_*DNA*_, where each state is characterized by a position-specific probabilistic parameter. Assuming a Markov chain of order *k*, the likelihood of a sequence given the model is:

(1) $$P\left(s_{1},s_{2},.........,s_{l}\right)=\prod_{i=1}^{l}P_{i}\left(s_{i}|s_{i-1}\right),$$

Where, the Markovian probability *P*_*i*_(*s*_*i*_) = *P*(*s*_*i*_|*s*_*i*-1_, *s*_*i*-2_,...., *s*_*i*-*k*_) denotes the conditional probability of a nucleotide at location *i *given the *k *predecessors. In the current application we use a first order Markov model to model the sequences and hence, *k *= 1.

### Information content

Information content of splice sites was calculated based on Shannon's information theory [28]. Entropy Shannon defined the information in an event *i*, to be -log *p*_*i *_where, *p*_*i *_is the probability that the event *i *occurs. The information contained in a splice site can be computed by summing up the information contents (*R*_*i*_, *bits*)of given nucleotides from individual positions, using the weight matrix generated from the frequency of each nucleotide at each position [29,30]. The individual information content of each individual splice site was calculated using the following equation [29,30]:

(2) $$R_{sequence}(l)=2+\sum_{b\in A,C,G,T}f(b,l)\times \log_{2}f(b,l)$$

where, *f*(*b*, *l*) is the probability of base *b *at position *l*.

We first generated an individual information weight matrix from the frequencies of each nucleotide at each position to calculate the information content (*R*_*i*_, *bits*) of each splice site sequence. The individual information weight matrix can be calculated by the following equation [29]:

(3) *R*_*iw*_(*b*, *l*) = 2 + log_2 _*f*(*b*, *l*)

The information content of each splice site was calculated by summing up *R*_*iw*_(*b*, *l*) at each position of the splice site sequences. The relationship between *R*_*iw*_(*b, l*) and *R*_*sequence *_(*l*) is provided in Additional file 2.

### Shapiro's score

Shapiro *et al. *[31] proposed a method to score the strength of splice sites based on percentage of each nucleotide at each position. First they create a frequency matrix of nucleotides in each of the positions of the splice site sequence. Shapiro's score for acceptor splice site is given by the equation [31]:

(4) *SS*_*acceptor *_= 100*((*t*1-*l*1)/(*h*1-*l*1) + (*t*2-*l*2)/(*h*2-*l*2))/2

where, *t*1 is the sum of best 8 of 10 nucleotide percentages at position -13 to -4

*l*1 is the sum of lowest 8 of 10 nucleotide percentages at position -13 to -4

*h*1 is the sum of highest 8 of 10 percentages at position -13 to -4

*t*2 is the sum of best nucleotide percentages at position -3 to + 1

*l*2 is the sum of lowest nucleotide percentages at position -3 to + 1

*h*2 is the sum of highest nucleotide percentages at position -3 to +1

Similarly, Shapiro's score for donor splice site is given by the equation [31]:

(5) *SS*_*donor *_= 100*(*t *- min)/(max - min)

where, *t *is the sum of percentages at position -3 to + 7

min is the sum of lowest percentages at position -3 to + 7

max is the sum of highest percentages at position -3 to + 7

### Sequence feature elimination based on F-score

Sequence feature elimination is an important step towards the classification task. Classifiers like neural networks, SVM's etc. perform better when they are trained with meaningful input data. Redundant data often causes misclassification and hence, the reduction of classification performance. So it is desirable to eliminate the less important features from the input data and to select those features that can potentially discriminate between true and false class. According to Dror *et al. *[32], there are three potential benefits of feature selection: improving the performance of the classifier, producing a cost-effective classifier, and providing a better understanding of the problem.

In this work, we select most informative acceptor and donor splice site features, and we used the F-score feature selection criteria also employed by Golub *et al. *[33] and Dror *et al *[32]. For each feature *x*_*j*_, *j *= 1, 2,...., *N*, we calculate the mean $\mu_{j}^{+}$ (for positive/true class) and $\mu_{j}^{-}$ (for negative/false class), standard deviation $\sigma_{j}^{+}$ (for positive/true class) and $\sigma_{j}^{-}$ (for negative/false class). The F-score *F*(*x*_*j*_) is calculated by:

(6) $$F\left(x_{j}\right)=\left|\frac{\mu_{j}^{+}-\mu_{j}^{-}}{\sigma_{j}^{+}-\sigma_{j}^{-}}\right|$$

### Support vector machine

The SVM is a statistical machine learning algorithm initially proposed by Vapnik [34-37] and applied to a wide range of pattern recognition problems [9,12,15,35,37,38]. It uses a hypothetical space of linear functions in a high dimensional feature space trained with a learning algorithm based on optimization theory. A SVM selects a small number of critical boundary samples (known as support vectors) from each class and builds a linear discriminant that separates them as widely as possible. In the case that no linear separation is possible, the 'kernel' technique is applied to map the training samples into a higher-dimensional space, and to learn a separator in that space [39]. SVM classification is an optimization problem given by:

(7) $$\text{Maximize }L=\sum_{i=1}^{l}\alpha_{i}-\frac{1}{2}\sum_{i,j=1}^{l}\alpha_{i}\alpha_{j}y_{i}y_{j}\text{K}\left({\text{x}}_{\text{i}},{\text{x}}_{\text{j}}\right),$$

(8) $$\begin{array}{cc} \text{s}.\text{ t}. & \sum_{i=1}^{l}\alpha_{i}y_{i}=0 \end{array}$$

(9) 0 ≤ *α*_*i *_≤ *C*, *i *= *1*,..., *l*,

where, *l *is the number of training examples, *K *is the kernel function, ***x ***is the input vectors, *y *is either -1 or +1 representing two different classes, *α *is the variable to be optimized and *C *is a trade-off parameter for generalization performance [35,36]. Each *α *corresponds to one particular training example and after the training process, only a subgroup of *α *will have non-zero values. This subgroup of *α *and their corresponding training examples are called the support vectors. In this study, two separate SVM classifiers are required, one for acceptor and one for donor. The class labels *y *in the two classifiers would then indicate true (*y *= +1) or false sites (*y *= -1) for acceptor and donor accordingly. Given a query DNA segment *z*, the trained SVM classifies based on the decision function:

(10) $$o\left(z\right)=\text{sign}\left[\sum_{i\in \nu }\alpha_{i}y_{i}K\left(x_{i},z\right)\right],$$

where *v *is the set of support vectors.

### Dataset

To evaluate the performance of the proposed models, we used publicly available NN269 [10] splice site dataset. The dataset is divided into two groups namely: the acceptor splice sites and the donor splice sites. It contains 1324 confirmed true acceptor splice sites, 5552 false acceptor sites, 1324 confirmed true donor sites, and 4922 false donor sites collected from 269 human genes. The pseudo or false acceptor/donor splice sites are those having AG/GT in the splicing junction but not a real acceptor or donor splice site according to the annotation. Acceptor splice sites have a window of 90 nucleotides (-70 to +20) with the consensus nucleotides AG at positions -69 and -70. This window includes the last 70 nucleotides of an intron and the first 20 nucleotides of the succeeding exon. On the other hand, donor splice sites have a window of 15 nucleotides (-7 to +8) with the consensus nucleotides GT at positions +1 and +2. This window includes the last 9 nucleotides of an exon and the first 6 nucleotides of the succeeding intron. The acceptor and donor splice site datasets are divided into a unique training and test dataset. The test datasets do not contain any sequence which is in training dataset. The training dataset contains 1116 true acceptor, 1116 true donor, 4672 false acceptor, and 4140 false donor sites. The test data set contains 208 true acceptor sites, 208 true donor sites, 881 false acceptor sites, and 782 false donor sites.

### Model learning

The learning of the model is designed in two phases. Phase one consists of estimation of Markov parameters, scoring information content of sequences, and calculation of Shapiro's score. In phase two SVM is trained with polynomial and Gaussian kernels.

All the training sequences were aligned with respect to the consensus sequence for the estimation of the Markov parameters. We only use the true training sequences to create the Markov model. The estimates of the MM1 are the ratios of the frequencies of each dinucleotide in each sequence position as shown in the following equation [18].

(11) $${\hat{P}}_{i}\left(s_{i}\right)=\frac{\#\left(s_{i-k}^{i}\right)}{\#\left(s_{i-k}^{i-1}\right)},$$

**Figure 5:**
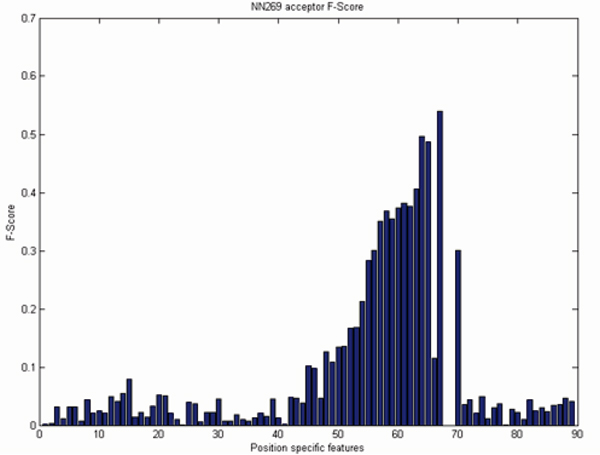
F-Score analysis of NN269 acceptor splice site.

**Figure 6:**
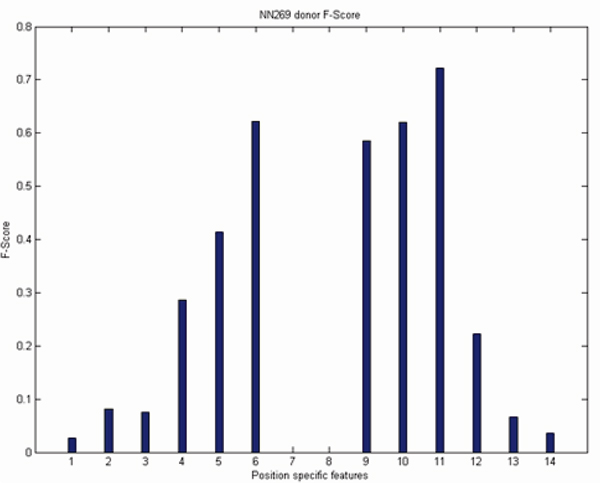
F-Score analysis of NN269 donor splice site.

For a sequence of length n there are n-1 position specific probabilistic parameters [18]. As the length of the acceptor splice site is 90 nucleotides there are 89 MM1 parameters and for 15 nucleotide long donor splice site there are 14 MM1 parameters. We reduce the size of the MM1 parameters based on the F-score. We empirically selected the F-score value 0.20. There are many inputs with an f-score value less than 0.20 as shown in Figure 5 and 6. However, their inclusion did not significantly improve the performance and increased the computational complexity and training time. As the f-score shows the position-specific discrimination between true and false splice sites, a higher F-score value indicates a better discrimination between true and false splice sites and conveys more information to the SVM. Based on the F-score values, position specific MM1 parameters are reduced from 89 to 19 for acceptor splice sites and from 14 to 9 MM1 parameters for donor splice sites. Based on the above discussion we propose several models which are listed in Table 5.

A position specific nucleotide background matrix is required for the calculation of information content and Shapiro's scores. A generalized frequency matrix of the splice site regions of the whole genome is preferable as it gives the most reasonable statistics of the occurrence of nucleotides in the splice site. However, we only use the NN269 true training data to construct the frequency matrix. As we compared the performance of MM1 SVM [18] with that of IC Shapiro SVM, it is required that the same training data be used to create MM1 parameters, information content and Shapiro's scores. To calculate the information content score the individual information content weight matrix *R*_*iw *_(*b*, *l*) is created from the nucleotide background matrix following equation (2) (refer to the method section). Then the information content is calculated by summing up *R*_*iw *_(*b*, *l*) of the specified positions. Similarly the nucleotide background matrix is used to calculate the Shapiro's score for acceptor and donor splice sites following equations (3) and (4) respectively.

We used the leave one out cross validation technique is applied to determine the splice site prediction accuracy and to compare the predictive accuracy with other methods. The cross validation is performed by randomly partitioning the data into five independent subsets. Each of the subsets does not share any repeating sequences. Each model was trained by selecting four of the subsets (training data) and was tested on the remaining one. Finally, we took the average of the five prediction accuracies as the final prediction measure of the model.

### Performance measures

The classification performance of the models is measured in terms of their sensitivity (*S*_*N*_), and specificity (*S*_*P*_). Sensitivity, also known as true positive rate (TPR), is defined as the percentage of correct prediction of true sites while specificity is the correct prediction of false sites as defined below:

$$\begin{array}{cc} Sensitivity\left(S_{N}\right)=\frac{TP}{TP+FN} & Specificity\left(S_{P}\right)=\frac{TN}{TN+FP} \end{array}$$

Where, TP, TN, FP, and FN stand for true positive rate, true negative rate, false positive rate, and false negative rate. They are defined in Table 6[40].

**Table 6:** Definition of TP, TN, FP and FN

	Predicted positive	Predicted negative
Real positive	true positives, TP	false negatives, FN

Real negative	true negatives, TN	false positives, FP

Also receiver operator curve (ROC) is drawn using the sensitivity and specificity values. ROC analysis is an effective and widely used method for assessing the classification performance [40]. When a ROC is created from the sensitivity (the y axis) and specificity (the x axis) of a model, the closer a curve follows the left-hand border and then the top of the border of the ROC plot, the more accurate the model is (refer to Figure 3, 4, 5 and 6). We also calculate the area under ROC curve (AUC), as classification performance of some of the models are very close and may not clearly distinguish performance of two models when we view them in the ROC curve. However, AUC accurately measure the total ROC area covered by a model.

## Competing interests

The authors declare that they have no competing interests.

## Authors' contributions

AKMAB provided the conception and design of this study, the implementation of the method and its analysis. BC and SKH contributed to the design of the study and the interpretation of the results. All authors contributed to the writing and critically revising the manuscript.

## Supplementary Material

Additional file 1 AUC and SVM parameters for different models for NN269 acceptor and donor splice sites. Click here for file

Additional file 2 Relationship between *R*_*iw *_(*b*, *l*) and *R*_*sequence *_(*l*). Click here for file
