# Supplementary material for: Fast splice site detection using information content and feature reduction
Source: BMC Bioinformatics. 2008 Dec 12;9(Suppl 12):S8. doi: 10.1186/1471-2105-9-S12-S8 (PMC2638148; doi:10.1186/1471-2105-9-S12-S8)
Supplement: Additional file 1 — AUC and SVM parameters for different models for NN269 acceptor and donor splice sites. [file 1471-2105-9-S12-S8-S1.pdf]

Additional file 1

Table S1

AUC and SVM parameters for different models for NN269 acceptor splice sites

**Model**	**SVM kernel**	**AUC**	**SVM Parameters**
Reduced MM1 SVM<br>(Best in terms of accuracy)	GRBF	**0.9741**	Sigma = 2, C=10
Reduced MM1 SVM	Polynomial	0.9695	Order = 2, C=10
MM1 SVM [18]	Polynomial	0.9674	Order = 2, C=10
MM1 SVM	GRBF	0.9695	Sigma = 1, C=10
IC Shapiro SVM<br>(Best In terms of Time)	Polynomial	**0.9628**	Order = 2, C=10

AUC and SVM parameters for different models for NN269 donor splice sites

**Model**	**SVM kernel**	**AUC**	**SVM Parameters**
Reduced MM1 SVM<br>(Best in terms of accuracy)	GRBF	**0.9790**	Sigma = 1, C=10
Reduced MM1 SVM	Polynomial	0.9764	Order = 2, C=10
MM1 SVM [18]	Polynomial	0.9761	Order = 2, C=10
MM1 SVM	GRBF	0.9780	Sigma = 1, C=10
IC Shapiro SVM<br>(Best In terms of Time)	Polynomial	**0.9665**	Order = 2, C=10
