# Supplementary material for: Fast splice site detection using information content and feature reduction
Source: BMC Bioinformatics. 2008 Dec 12;9(Suppl 12):S8. doi: 10.1186/1471-2105-9-S12-S8 (PMC2638148; doi:10.1186/1471-2105-9-S12-S8)
Supplement: Additional file 2 — Relationship between Riw (b, l) and Rsequence (l). [file 1471-2105-9-S12-S8-S2.pdf]

Additional file 2

Additional text – Relationship between $R_{iw}(b,l)$ and $R_{sequence}(l)$:

The individual information content of each individual splice site was calculated using the following equation [29, 30]:

$$R_{sequence}(l) = 2 + \sum_{b \in A,C,G,T} f(b,l) \log_2 f(b,l) \qquad (2)$$

where, $f(b,l)$ is the probability of nucleotide b at position l. To calculate the information content $(R_i, bits)$ of each splice site, at first, an individual information weight matrix is generated from the frequencies of each nucleotide at each position. The individual information weight matrix $R_{i,w}(b,l)$ can be calculated from the following equation [29, 30]:

$$R_{i,w}(b,l) = 2 + \log_2 f(b,l) \qquad (3)$$

The information content of each splice site was calculated by summing up $R_{i,w}(b,l)$ at each position of the splice site sequences. The relationship between $R_{i,w}(b,l)$ and $R_{sequence}(l)$ is discussed below.

In a set of aligned sequences the jth sequence is represented by a matrix $s(b,l,j)$, where b is the base at position l. The individual information of sequence j is the dot product between the sequence and the weight matrix $R_{i,w}(b,l)$ as given below:

$$R_i(j) = \sum_l \sum_{b=A}^{T} s(b,l,j) R_{iw}(b,l) \qquad (4)$$

The frequency matrix $f(b,l)$ is created by aligning n individual sequences and given by:

$$f(b,l) = \frac{1}{n} \sum_{j=1}^{n} s(b,l,j) \qquad (5)$$

As the frequencies also sum to one, we can write:

$$\sum_{b=A}^{T} f(b,l) = 1 \qquad (6)$$

The mean information content of n sequences used to create the frequency matrix $f(b,l)$ is given by:

$$E(R_i) = \frac{1}{n} \sum_{j=1}^{n} R_i(j) \qquad (7)$$

From equation (3) and (4) we get:

$$R_i(j) = \sum_l \sum_{b=A}^{T} s(b,l,j)[2 + \log_2 f(b,l)] \qquad (8)$$

From equation (7) and (8) we get:

$$E\left(R_i\right)=\frac{1}{n}\sum_{j=1}^{n}\sum_{l}\sum_{b=A}^{T}s\left(b,l,j\right)[2+\log_2 f\left(b,l\right)] \qquad (9)$$

From equation (5) and (9) we get:

$$E\left(R_i\right)=\sum_{l}\sum_{b=A}^{T}f\left(b,l\right)[2+\log_2 f\left(b,l\right)] \qquad (10)$$

$$=\sum_{l}\sum_{b=A}^{T}2f\left(b,l\right)+\sum_{l}\sum_{b=A}^{T}f\left(b,l\right)\log_2 f\left(b,l\right)]$$

From equation (6) and (10) we get:

$$E\left(R_i\right)=\sum_{l}2+\sum_{l}\sum_{b=A}^{T}f\left(b,l\right)\log_2 f\left(b,l\right)] \qquad (11)$$

The right hand side of equation (11) is similar to that of $R_{sequence}\left(l\right)$ in equation (2). Hence, it can be deduced that the average of individual information content is the average information content of the sites.
